# Quality of life of patients with fibrodysplasia ossificans progressiva

**DOI:** 10.1007/s11832-015-0704-6

**Published:** 2015-11-13

**Authors:** Fernando Ortiz-Agapito, Douglas Colmenares-Bonilla

**Affiliations:** Hospital Regional de Alta Especialidad del Bajio, Boulevard Milenio No. 130, Col. San Carlos la Roncha, C.P. 37660 León, Guanajuato Mexico

**Keywords:** Fibrodysplasia ossificans progressiva, Quality of life, FOP, SF36

## Abstract

**Introduction:**

Fibrodysplasia ossificans progressiva (FOP) is a rare disorder characterized by episodes of acute pain and heterotopic ossification of soft tissue, and progressively limited physical function and social participation.

**Objective:**

We aimed to determine the impact of FOP on quality of life, specifying areas or dimensions most affected.

**Materials and methods:**

This was a transverse observational study; patients with FOP were assessed using the Short Form 36. Questionnaire results were obtained using Quality Metric software and analyzed using frequency distribution, percentages and measures of central tendency.

**Results:**

Eight patients, mean age 30.2 years, were included. The physical dimension was the most affected, with an average of 25.5 points. The most representative items were impaired function and physical role. Physical pain was found with an average of 44.5 points. The best scores were reported in the areas of emotional role and mental health, with an average of 79 and 76 respectively.

**Conclusions:**

FOP is a severely disabling disease, generating a significant deterioration in quality of life secondary to progressive deterioration in physical abilities. The findings of this study demonstrate good self-rated health of participants.

## Introduction

Fibrodysplasia ossificans progressiva (FOP), also known as myositis ossificans progressiva, is a rare disorder with an estimated incidence of 1 in 2,000,000 [1]. It is the most severe cause of disabling ossification disease in humans [2]. The inheritance is autosomal dominant, though most cases are de novo mutations [3]. The etiological basis is presumed to be a deregulation of bone morphogenetic proteins, due to an alteration in the *ACVR1* gene [4].

The clinical picture is characterized by episodes of acute pain, followed by the appearance of subcutaneous nodules (flare-ups) that evolve to ossify the soft tissues (muscles, ligaments, fascia, tendons), with a cephalo-caudal pattern, starting in the dorsal, axial, cephalic and proximal areas, progressing to ventral, appendicular, caudal and distal tissues [5]. The progression of this cascade leads to multiple and progressive ossification forming an exoskeleton in the patient [6].

The cycle trigger may be low-energy trauma, intramuscular injections, simple contusions or viral infections, among other stress factors [7].

**Fig. 1:**
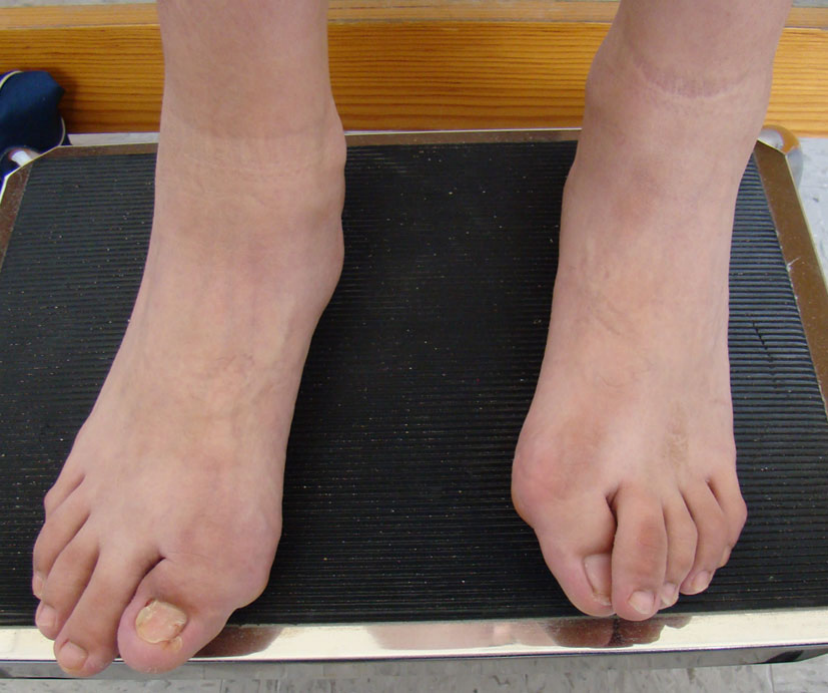
Clinical image of the main suspect characteristic in FOP: symphalangism with shortening and varus in toes

**Fig. 2:**
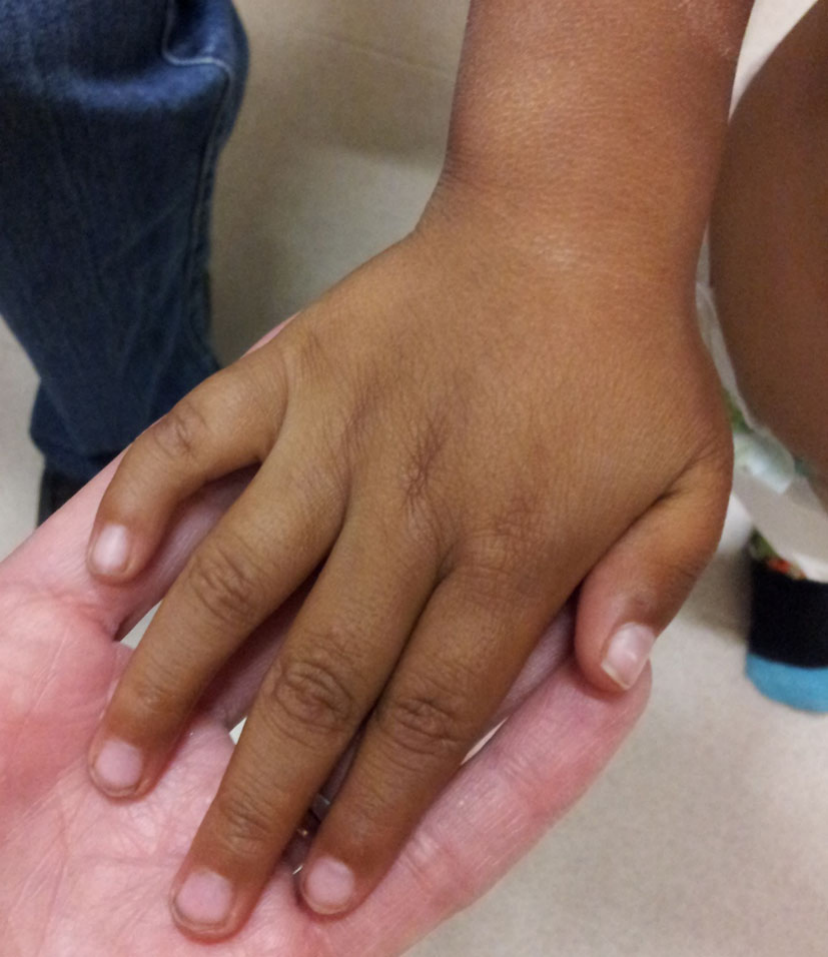
Shortening of thumb or first toes is the first sign in the FOP patient

**Fig. 3:**
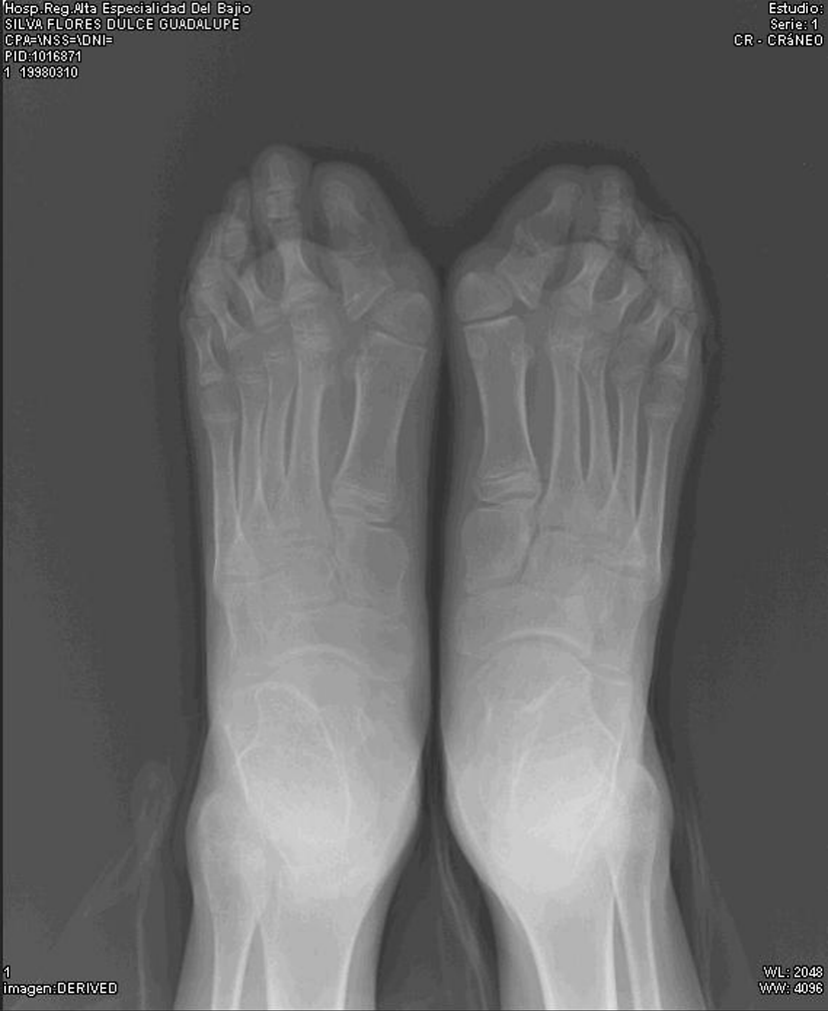
The varus and shortening in the same patient is caused by aberrant proximal delta phalange in the first toe

The diagnosis is purely clinical and pivot data is considered to be symphalangism with shortening and hallux varus [8], as shown in Figs. 1, 2 and 3.

**Fig. 4:**
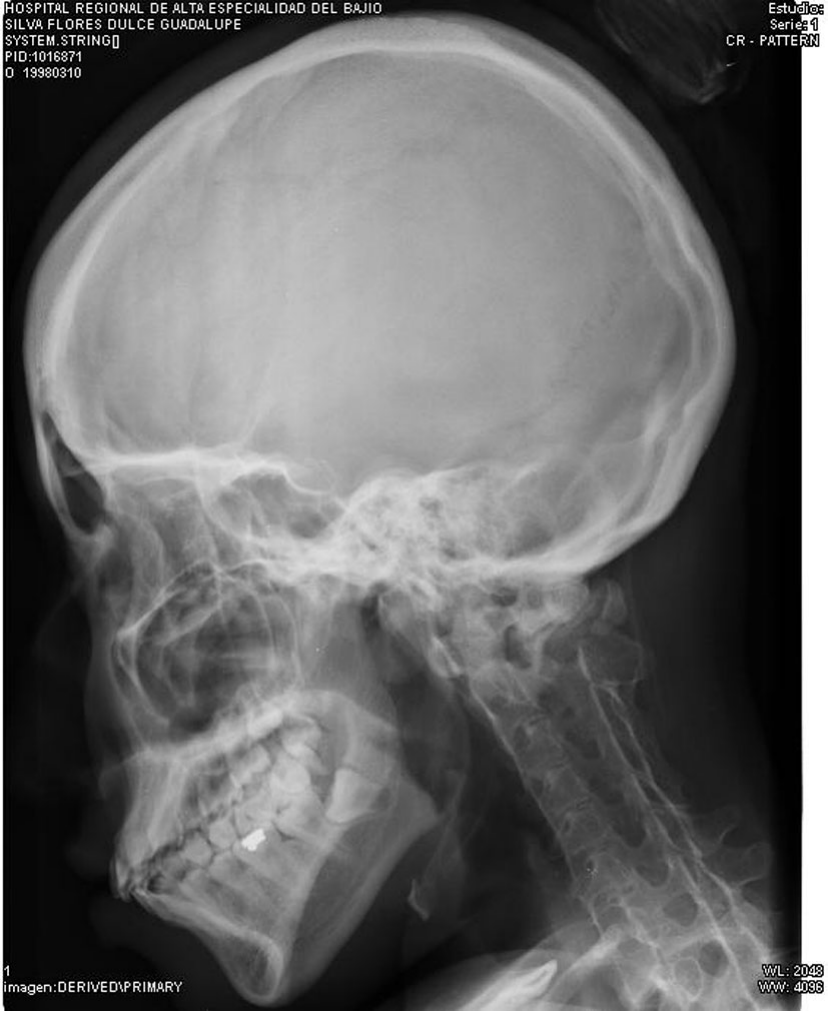
Characteristic pattern of ossification in FOP, showing progressive fusion of the cervical spine with decreasing range of movement

**Fig. 5:**
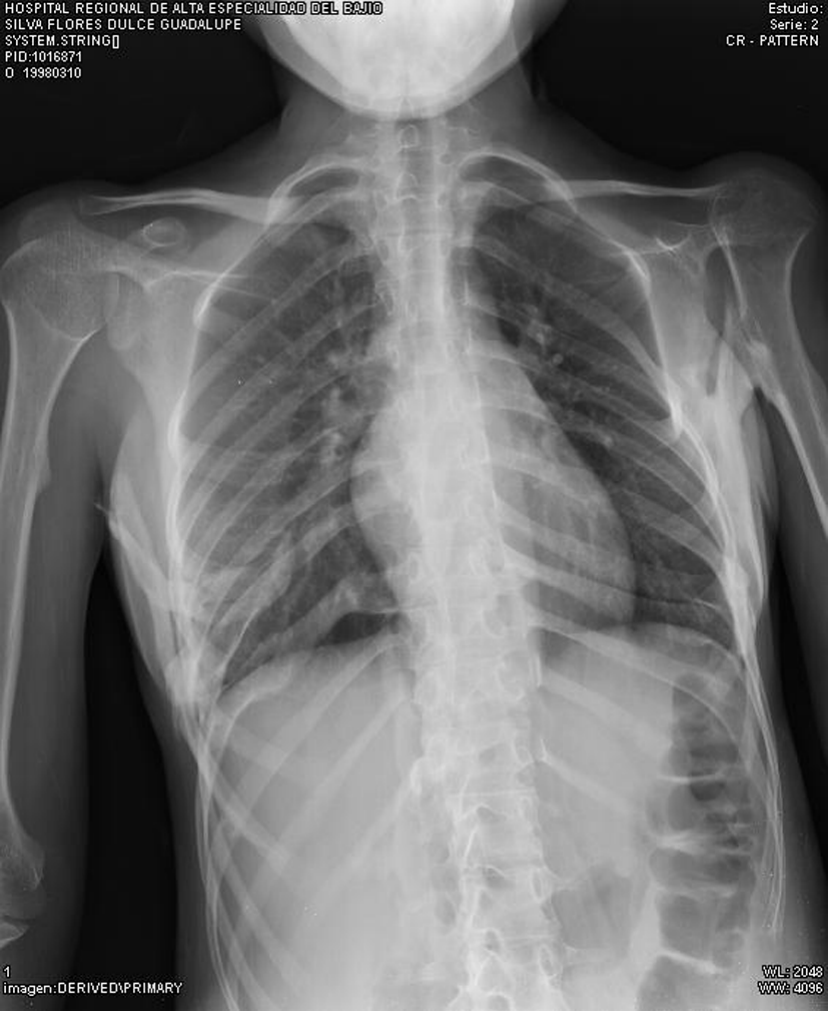
Bony bridges between scapula and left humerus, the right bridge developing. Ossification in the lowest right ribs

Misdiagnosis is often the rule because of the very low incidence of this disease. Patients generally undergo unnecessary procedures (biopsies, osteotomies, chemotherapy) that can lead to permanent disability in up to 50 % [9], accelerating the emergence of new nodules which ends in the cascade of nodules, ossification and progressive limitation that leads to permanent ossifications and disability [10] (Figs. 4, 5).

It is believed that the quality of life in patients with these characteristics is poor [11]; however, there are no studies in this field due to the low incidence of FOP. Most of the clinical papers published are case reports [12, 13].

There are in the literature different tools for assessing quality of life and degree of disability in patients with orthopedic disorders.

The Medical Outcomes Study 36-item Short Form (SF-36) [14] is a generic questionnaire that calculates the quality of life related to health in orthopedic and medical conditions. It may be reliably self-administered by the patient. It rates different aspects (physical functioning, physical and emotional role, pain and emotional health) so that the clinician can make an assessment for each domain independently or in general.

The aim of this study is to determine the impact of FOP on quality of life, describing the most affected areas, in order to decide on palliative care for these patients, since FOP is not yet a curable disease.

## Materials and methods

A cross-sectional study was developed comprising eight patients diagnosed with fibrodysplasia ossificans progressiva, maintaining contact or control in the Pediatric Orthopedic service at the High Specialty Regional Hospital in Bajio. All of them were of Latin American origin, so the Validated Spanish version of the SF-36 clinical questionnaire was used to assess the quality of life. All the individuals were asked whether or not they would participate in the study. All the forms were sent and the results compiled electronically. Assistance was given by evaluator through telephone or electronic monitoring.

The results of the questionnaires were obtained by online software Quality Metric^®^, scoring each item from 0 to 100. The weighted overall scores established standards and physical and mental dimensions.

Data analysis of qualitative variables was by frequency distribution and percentages. Quantitative variables were analyzed with measures of central tendencies (mode, median and mean).

## Results

### Clinical characteristics

Eight patients (six female and two male) were evaluated. Their ages ranged from 16 to 43 years, with a mean of 30.2 years. The demographics of the eight patients are summarized in Table 1. The values are broken down by category in Table 2.

**Table 1:** Sociodemographic characteristics of the study sample, *N* = 8

Values	Number	Percentage	Average
Sex			
Male	2	25	
Female	6	75	
Age (years)			30.2
Marital status			
Single	5	62.5	
Married	3	37.5	
Occupation			
Employed	4	50	
Unemployed	2	25	
Other	2	25	
Studies			
Elementary school	2	25	
High school	3	37.5	
College–University	3	37.5

**Table 2:** Descriptive questionnaire by category in SF-36

Results of SF-36	Patient no.							
	1	2	3	4	5	6	7	8
Physical function (PF)	0.0	55.0	35.0	5.0	40.0	35.0	35.0	0.0
Role physical (RP)	0.0	25.0	0.0	0.0	100.0	0.0	0.0	0.0
Body pain (BP)	12.0	41.0	62.0	10.0	72.0	41.0	32.0	84.0
General health (GH)	30.0	35.0	45.0	20.0	92.0	40.0	52.0	100.0
Vitality (VT)	60.0	60.0	60.0	50.0	90.0	80.0	25.0	65.0
Social function (SF)	50.0	37.5	62.5	25.0	100.0	37.5	37.5	75.0
Role emotional (RE)	100.0	100.0	100.0	33.3	100.0	100.0	0.0	100.0
Mental health (MH)	96.0	68.0	72.0	76.0	100.0	80.0	40.0	80.0

Mental health and emotional role are the most preserved values among FOP patients

The age at definitive diagnosis of FOP ranged from 4 to 29 years (mean 17.6 years), but the onset of symptoms started from 3 to 6 years in all patients with a cervical tumor or lump resembling infarcted ganglia, which was assumed in most cases to be a tumor. Only one of the patients had an early FOP diagnosis (4 years old), while the others were misdiagnosed with tumor, multiple ostheochondromatosis or myositis. Six of them were subject to more than four (range 4–16) invasive procedures (skin and bone biopsies, osteotomies) until a definite diagnosis was reached.

Tumor biopsy was performed in all eight patients, followed by more ossified lesions leading to progressive disability because of ossification, following a cephalo-caudal and centrifugal pattern. Clinical features present at birth in all patients were symphalangism of the great toe, all were diagnosed as congenital hallux varus. No other clinical or laboratory characteristic was present from birth.

At the time of the study, none of the patient had any marfanoid habitus, since their heights ranged from 1.50 to 1.70 m (mean 1.60 m).

Since the gold standard for diagnosis are clinical and radiographic features, only one of the patients had DNA analysis to confirm the classical mutation. There is no evidence that FOP patients have any increase in serum bone markers (alkaline phosphatase, serum calcium, lactic dehydrogenase, etc.) and these were not evaluated for this study.

### Functional results

Zero values were reported in two patients (34 and 43 years old), as they use wheelchairs for mobility, reporting themselves as nearly fully dependent for feeding, self-cleaning and transportation. The highest value in this category came from a 26-year-old female who does not need any assistance in walking. In no case did values reach the minimum mean value for the normal population, which shows the severe effect on physical function in FOP patients.

Regarding the pain scale, values ranged from 10 to 84 points. The lowest value (most pain) was from a 17-year-old female who was going through an active phase of the disease, followed by a 34-year-old male who has almost full body immobility. The highest value (least pain) came from a 32-year-old female who is fully functional and ambulatory.

Emotional role does not seem to be affected. Every patient showed a solid emotional status (except for a 17-year-old female who was undergoing an active phase of the disease).

Mental health was not dependent on time from onset of symptoms or years lived with this condition, since high values were obtained in patients with more than 20 years of the disease. The lowest score was from a single 30-year-old male without any other external factor.

### Individual values

Regarding the individual results, the best global score (85 points) was achieved from the individual with the highest mental, emotional and social scores; this is mainly because she is a very independent single woman who does not need any assistance for activities of daily living.

The lowest score (27.4 points) was recorded in the young female who was living through an active phase of the disease, and was restricted in mobility and manifested difficult pain control.

In the rest of the patients (all of them in a quiescent phase of disease), the lowest global score was 27.6 points in one individual with a worsening level of independence.

The worst outcome (in patients who were in a quiescent phase of disease) was seen in one 28-year-old male who has lived with the disease the longest (28 years from beginning of symptoms).

Scores on the physical and mental dimensions are summarized in Table 3.

**Table 3:** Summary of scores on physical and mental dimension SF-36

Summary scales	Patient no.							
	1	2	3	4	5	6	7	8
Physical Component Summary (PCS)	8.5	29.3	26.5	14.2	41.9	22.5	32.7	28.9
Mental Summary Score(MCS)	68.4	51.7	57.7	49.8	68.3	60.1	30.2	64.1

The severe physical impairment of all participants is clear since the higest value (best result) must approach 100 points

It is noted that the physical dimension is the most affected, presenting the worst scores with a mean of 25.5. The most representative data show deterioration in function and physical role. Pain is one of the primary characteristics of this disease and was found with an average of 44.25 points.

Of the four main scales, representative examination of mental health and social function had lower correlations. The best scores were reported in the areas of emotional role and mental health with an average of 79 and 76 points, respectively.

## Discussion

There is poor functional outcome of patients with FOP from severe mobility restriction as they progress in the disease. It is to be expected that older patients show lower scores on function and physical role, and those who are younger, even though they preserve more mobility, do not have a high enough score to be considered independent (maximum of 55 points in subject 2). One would expect that with more progress in functional limitation all scales would be affected; however, this did not occur in the emotional role, where high scores persisted. It is noteworthy that no patients were receiving psychological therapy that helped them to accept or cope with their situation.

## Conclusion

FOP is an extremely rare disease; subjects who suffer from it still undergo multiple procedures that aggravate their condition before the correct diagnosis [15]. To date, there is no treatment, and the only demonstrably effective medical intervention is the application of high-dose steroids within the first 24–36 h of the initial lesion (flare-up) [16]. Progression towards limiting basic activities and hygiene is so severe that around 30 years old, most patients are confined to a wheelchair with consequent burden and and strain on the family, both socially and economically [17].

The mean life expectancy is 50–60 years as a result of cardiopulmonary complications and severe malnutrition [18].

To the best of our knowledge there is no other study on the general functional status of patients with this disease.

The present study has several limitations, especially the size of the sample; however, the low incidence of this pathology makes a larger study difficult. Studies must be larger and continue to spread knowledge of this disease to avoid under-diagnosis and foster early recognition of these patients, in order to avoid a rapid progression to dependence in activities of daily living, since the quality of life in patients with FOP is initially reduced by physical mobility.

Using data collection instruments that measure and characterize the multidimensional health approach promises improvements in the doctor–patient relationship, so that patients will have a new framework regarding their health care providers, and the latter will be able to judge the effectiveness of management of the population served.
